# Transactions of the American Association of Obstetricians and Gynecologists

**Published:** 1889-07

**Authors:** 


**Transactions of the American Association of Obstetricians and Gynecologists.** Vol. I. For the year 1888. Illustrated; 8vo; pp. xxxviii., 347. Philadelphia: William J. Dornan, printer. 1888. Cloth, $5.00; half Russia, $6.00. Buffalo: Peter Paul & Bro.

The great number of National special societies, and the vast literature they annually place at the disposal of the profession, makes it necessary to scrutinize carefully the quality of the work done by any new candidate for recognition and favor. The volume in question is not, it must be confessed, a disappointment, whether judged by the standard set up by its older and larger companions, or by the more searching eye of the critic seeking for inherent worth in the material offered by its contributors.

A synopsis of the proceedings of the Association having been published in the JOURNAL for October, 1888, it will only be necessary to speak here of some matters of particular importance, that could not be elaborated at that time.

The papers of Drs. Price and Cushing, involving the question of drainage in abdominal and pelvic surgery, and the discussion that followed them, brought out in strong light the salients ot this subject, and merit careful study in view of the results obtained.

The surgical treatment of the peritoneum for suppurative inflammation receives intelligent and satisfactory handling by Drs. Myers and Montgomery in two admirable papers. This is one of the live subjects in the field of abdominal surgery, and one that demands the serious study of the general practitioner; for he must be able to early recognize the necessity for surgical interference, and to adequately provide therefor, if he does not operate himself.

Dr. Vanderveer's paper on the Relation of the Abdominal Surgeon to the Obstetrician and Gynecologist is a philosophic presentation of an important phase of specialism, and will serve to strengthen the hands of the practiser of abdominal surgery in his dealings and intercourse with the others. This paper ought to be in the hands of every general practitioner.

The perineum, both in its obstetrical and surgical relations, has been written and talked about, until it would seem that nothing new remained to be presented about this peninsular body that so often projects a woman into a sea of trouble. Yet Dr. Marcy has presented the whole subject, anatomically, physiologically, and surgically, in such a manner, and with so much new light on disputed points, and has, withal, illustrated the subject with so many original and useful plates, that one cannot but feel that this one paper is worth more than the price of the volume.

The Induction of Premature Labor, by Dr. Stanton, is a paper full of interesting material for study and deliberate thought; and Dr. Opie's classic, entitled, Is the Frequent Use of the Forceps Abusive? presents the other side of a picture that is too often seen only from the obverse.

The papers on Tumors of the Abdominal Walls, by Drs. Ill and Reed, deserve particular mention, not only on account of the sparsity of literature on the subject, but, again, because of the thorough, intelligent, and scholarly treatment it receives at their hands. The colored plates accompanying Dr. Ill's paper are something unique in surgical art-illustration, and deserve especial mention.

Other papers are entitled to particular notice on account of their merit and importance, but we are limited in our space, and must be content to pass them by at present with a mere mention of their titles. They are as follows:

Surgical Treatment for Laceration of the Perineum and Pelvic Floor, by W. H. Wathen, M. D.; Double Ovariotomy during Pregnancy—Subsequent Delivery at Term, by William Warren Potter, M. D.; Vaginal Tamponnement in the Treatment of Prolapsed Ovaries, by W. P. Manton, M. D.; Note on the Treatment of Endometritis by Injections of Pure Nitric Acid, by A. Cordes, M. D., Geneva, Switzerland; The Treatment of Puerperal Eclampsia, by the same author; A Contribution to the Study of the Reflex Neuroses of Pregnancy, with a Case of Aphasia Graviditatis, by G. A. Moses, M. D.; Intraperitoneal Hematocele, by J. H. Scarff, M. D.; An Unusual Case of Subserous Uterine Fibroid with Operation, by Hampton E. Hill, M. D.; Treatment of Certain Cases of Salpingitis, by A. P. Clarke, M. D.; The Influence of the Sexual Life of Woman in the Etiology of Certain Diseases of the Ear, by Thomas E. McArdle, M. D.; Diseases of the Skin associated with Disorders of the Female Sexual Organs, by George H. Rohé, M. D.; The Resuscitation of Asphyxiated New-born Infants by the Suspension Method, by Llewellyn Eliot, M. D.; Report of Sixty-three Cases of Alexander's Operation, by J. H. Kellogg, M. D.; Some Minute but Important Details in the Management of the Continuous Current in Gynecology, by A. Lapthorn Smith, M. D.; Hyaline Metamorphosis of the Placenta—Death of the Fetus in the Fourth Month, and Delivery of the Unbroken Ovum at Term, by Sarah E. Post, M. D., with a Pathological Demonstration by Wm. H. Welch, M. D.; Hysterectomy for Malignant Diseases of the Uterus, by William H. Wathen, M. D.; A Case of Removal of the Uterine Appendages—A Double Fallopian Tube, by Frank A. Glasgow, M. D.; The Diagnosis and Treatment of Uterine Fibroids, by Thomas J. Maxwell, M. D.; Heart Failure in the Puerperium, by Thomas Lothrop, M. D.; and, A New Operation for the Repair of the Perineum, by Bernard Burns, M. D.

The volume closes with a discussion on Extra-Uterine Pregnancy, participated in by eight or nine men of acknowledged worth, whose work in the field of abdominal surgery in general, and ectopic pregnancy in particular, entitle them to speak with the weight of authority. We have considered this subject somewhat *in extenso* of late in the columns of the JOURNAL, and need not repeat ourselves here. This discussion has been reprinted in a neat little brochure of sixty-seven pages, finely illustrated, that may be obtained for the small outlay of seventy-five cents, by any who desire some of the latest and best literature on this important, dangerous, and all too frequent accident of reproduction.

The paper, press-work, and binding of the book must command the admiration of every one who sees it, while the illustrations are superior, as a whole, to any we have seen in a volume of Transactions. A society that has launched its ship so successfully, deserves to be congratulated, for the voyage must surely be accomplished in safety.

